# Anti-Androgenic Therapies Targeting the Luminal Androgen Receptor of a Typical Triple-Negative Breast Cancer

**DOI:** 10.3390/cancers15010233

**Published:** 2022-12-30

**Authors:** Avinash Khadela, Vivek P. Chavda, Shruti Soni, Kaivalya Megha, Aanshi J. Pandya, Lalitkumar Vora

**Affiliations:** 1 Department of Pharmacology, L. M. College of Pharmacy, Navrangpura, Ahmedabad 380009, Gujarat, India; 2 Department of Pharmaceutics and Pharmaceutical Technology, L. M. College of Pharmacy, Ahmedabad 380009, Gujarat, India; 3 Pharm. D Section, L. M. College of Pharmacy, Navrangpura, Ahmedabad 380009, Gujarat, India; 4 School of Pharmacy, Queen’s University Belfast, 97 Lisburn Road, Belfast BT9 7BL, UK

**Keywords:** luminal androgen receptor, androgen receptor-positive, triple-negative breast cancer, anti-androgen therapy, enzalutamide, bicalutamide

## Abstract

**Simple Summary:**

The literature aims to explore anti-androgenic particulars against luminal androgen receptor-specific triple-negative breast cancer, thus providing a concise report on the clinical profile of anti-androgens. This review summarizes the types of triple-negative cancer cells, along with the brief involvement of androgen receptors in the luminal type. The paper intricately describes the evidence obtained from the clinical trials and confers the conclusions, in an attempt to help clinicians set forth the optimal strategies in mitigating LAR-TNBC and creating a steadfast foundation for patient care.

**Abstract:**

Triple-negative tumors are progressively delineating their existence over the extended spectrum of breast cancers, marked by intricate molecular heterogeneity, a low overall survival rate, and an unexplored therapeutic approach. Although the basal subtype transcends the group and contributes approximately 80% to triple-negative breast cancer (TNBC) cases, the exceptionally appearing mesenchymal and luminal androgen receptor (LAR) subtypes portray an unfathomable clinical course. LAR with a distinct generic profile frequently metastasizes to regional lymph nodes and bones. This subtype is minimally affected by chemotherapy and shows the lowest pathologic complete response. The androgen receptor is the only sex steroid receptor that plays a cardinal role in the progression of breast cancers and is typically overexpressed in LAR. The partial AR antagonist bicalutamide and the next-generation AR inhibitor enzalutamide are being assessed in standard protocols for the mitigation of TNBC. There arises an inevitable need to probe into the strategies that could neutralize these androgen receptors and alleviate the trajectory of concerning cancer. This paper thus focuses on reviewing literature that provides insights into the anti-androgenic elements against LAR typical TNBC that could pave the way for clinical advancements in this dynamic sphere of oncology.

## 1. Introduction

According to a World Health Organization (WHO) report, breast cancer (BC) affects 2.1 million women annually and accounts for the majority of women’s cancer-related fatalities [1,2]. The incidence and fatality rates of BC have significantly increased during the past three decades [3]. Different immunohistochemical characteristics enable the classification of this carcinoma into five subtypes, namely, luminal A, luminal B, human epidermal growth factor receptor-2 (HER2), triple-negative breast cancer (TNBC), and normal-like BC [4]. The prevalence of different subtypes of BC is listed below in decreasing order, with luminal A showing the maximum prevalence followed by TNBC, luminal B, HER2, and normal-like BC. The prognosis ranges from excellent for the luminal A type to the least favorable for TNBC [5]. TNBC delineates an intricate and diversified subtype of BC with an absence of estrogen receptor (ER), progesterone receptor (PR), and HER2 expression in the tumor microenvironment (TME). TNBC accounts for over 10–15% of BC cases, and its incidence is higher in premenopausal women. Moreover, it is disproportionately higher among Africans, with an influence on the psychological and financial aspects of the patients [6]. Since there is enormous heterogeneity within TNBC, it is subclassified into numerous categories. On the foundation of the gene expression portfolio, TNBC embodies six distinct subclasses, which include immunomodulatory (IM), luminal androgen receptor (LAR), basal-like 1 (BL-1), basal-like 2 (BL-2), mesenchymal (M), and mesenchymal stem-like (MSL). However, the recently revised classification involves just the IM, LAR, MES (mesenchymal), and BLIS (basal-like and immunosuppressed) [7,8].

The LAR subclass, representing 15–20% of TNBCs, is characterized by a modest prognosis and shows a minimal response to chemotherapy coupled with a lower complete response following neoadjuvant therapy. To differentially diagnose luminal from nonluminal tumors, a centroid model, namely, clustering among basal and luminal androgen receptors (CABAL), which embraces 426 differentially expressed genes in LAR and non–LAR TNBCs, was used [9]. The LAR subtype involves higher expression of genes associated with hormonally regulated pathways as well as genes linked with androgen receptors (ARs) and their coactivators [10]. The numerous proteins that are coactivators of AR interact with one or more domains of AR to enhance its effectiveness. Although some of the coactivator’s roles may be identical, several others have been studied extensively. Steroid receptor coactivator (SRC)-1, -2 and -3, members of the p160 group of coactivators, additionally enhance the action of insulin-like growth factor-1 and activate the Akt pathway to facilitate the activation of AR. By eliminating androgens, the transcriptional integrators p300 and cyclic AMP response element-binding protein (CBP) are upregulated, which may affect the antagonist/agonist ratio of nonsteroidal anti-androgens [11]. AR participates in the intricate process of cell signaling pathways and is an essential element in the development of breast tissues, maintaining an equilibrium in the proliferation of mammary cells [12]. To carry out gene expression, this steroid nuclear receptor forms a complex with testosterone and dihydrotestosterone in the cytoplasmic region of the mammary cell and advances toward the nucleus [13]. Androgens activate the AR and render positive or negative genetic regulations. Mutations that occur during these regulatory mechanisms threaten the occurrence of carcinogenesis [14]. The notable contribution of androgen-mediated growth stimulation and AR expression laid the foundation for androgen receptor antagonists as avant-gardes in the mitigation of LAR-associated TNBC [15]. AR antagonists extend a radical alternative to the management of chemoresponsive patients [16]. These entities exhibit their actions by acting as competitive inhibitors, thus ultimately ceasing the transcriptional activity of androgen receptors [17]. A meta-analysis was established to assess the efficacy of neoadjuvant chemotherapy (NAC) in the LAR and non-LAR subtypes. The NAC comprised taxanes in alternating combinations with doxorubicin, carboplatin, bevacizumab, veliparib and FEC regimens (fluorouracil, epirubicin and cyclophosphamide). This meta-analysis demonstrated the efficacy of NAC to be higher in the non-LAR setting than in the LAR subtype, which hints us toward failure of chemotherapy in mitigating LAR TNBC [15]. Thus, poor outcomes, decreased chemotherapeutic responsiveness, and a smaller pathologic complete response to chemotherapy necessitate the need for newer treatment strategies, leading to the exploration of anti-androgenic drugs [18]. Anti-androgenic molecules such as bicalutamide, enzalutamide, and abiraterone are better tolerated and have revealed remarkable efficacy, as evidenced by clinical studies in treating AR+ TNBC patients [19]. The objective of this review is to further explore the role of AR antagonists in AR-positive LAR-associated TNBC and to construct optimal literature for assessing their utilization in the oncological discipline.

## 2. TNBC Classification

**Figure 1:**
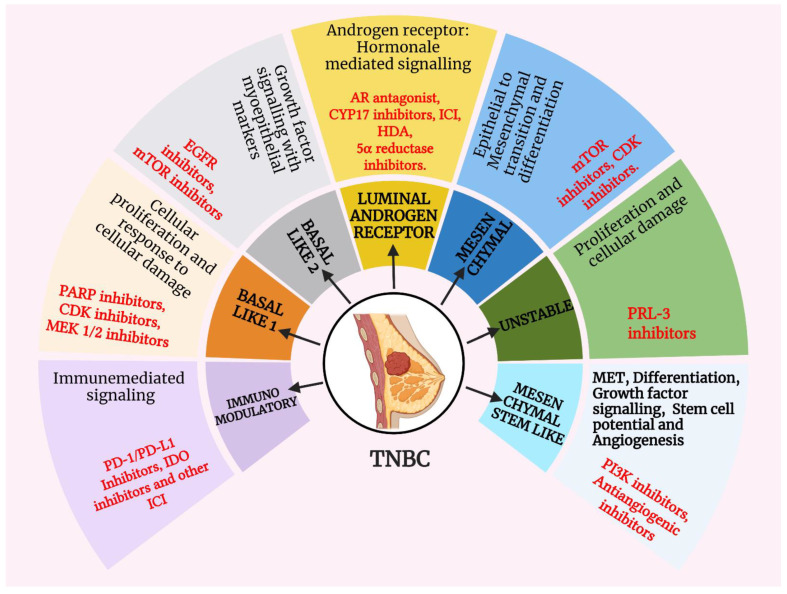
**Emphasizing the subtypical elements of TNBC.** Programmed cell death 1/programmed cell death ligand 1 (PD1/PD-L1), indoleamine 2,3-dioxygenase (IDO), immune checkpoint inhibitors (ICIs), poly adenosine diphosphate-ribose polymerase (PARP), cyclin-dependent kinase (CDK), mitogen-activated protein kinase (MEK), epidermal growth factor receptor (EGFR), mammalian target of rapamycin (mTOR), androgen receptor (AR), cytochrome P450 (CYP), phosphatase of regenerating liver-3 (PRL-3), phosphatidylinositol 3-kinase (PI3K), mesenchymal epithelial transition (MET).

Triple-negative cancer cells with a diversified nature embody subtypes with varied molecular characteristics. These distinctions at the molecular level render disproportionate responses to chemotherapeutic regimens [20]. The responses of tumor cells to chemotherapy, their pattern of recurrence, and prognosis were investigated to correlate with the apt recognition of the subtypes. Therefore, to develop meticulous and specified therapies against targetless TNBC tumor cells, we need to advance the identification of TNBC subtypes and their subtypical molecular hallmarks [21]. At the outset of an era in classifying triple-negative tumor cells, Kreike et al. concluded that typical TNBC is analogous to the basal class of breast cancers. Afterwards, numerous clinical studies suggested that TNBC has a heterogeneous essence and that ergo possesses distinct molecular characteristics that cannot be restrained precisely into the basal phenotype. Succeedingly, in 2011, Lehmann et al. submitted substantiations that supported the seven (stable-6; unstable-1) divisions of TNBC, namely, one unstable (UNS), two basal-like (BL1 and BL2), an immunomodulatory (IM) class, a mesenchymal-like (M) class, a mesenchymal stem cell (MSC) class, and finally a luminal androgen receptor (LAR) type [16,22]. Nevertheless, this classification had the paucity of a differential response to therapy, which led to further studies, and eventually, in 2016, FUSCC (Fudan University Shanghai Cancer Center) et al. set forth the four subdivisions of TNBC, including IM, LAR, MES and BLIS, following Burstein’s 2015 classification [8,23]. Several crucial biomarkers that aid in the diagnosis of such heterogeneous tumors are AR, forkhead box A1 (FOXA1), keratin 18 (KRT18), and X-box binding protein 1 (XBP1) for LAR-TNBC; Wnt, anaplastic lymphoma receptor tyrosine kinase (ALK), and transforming growth factor β (TGF-ß) for mesenchymal TNBC; epidermal growth factor receptor (EGFR), MET, and tumor protein P53 (TP53) for basal-like TNBC; and Janus kinase 1/2 (JAK1/2), signal transducer and activator of transcription 1/4 (STAT1/4), and tumor necrosis factor (TNF) for immunomodulatory TNBC [24]. To procure a wider understanding of the subtypes classified by different frameworks, we propose here the elucidations of all the existent subclasses of triple-negative tumors. Various subtypes of TNBC are diagrammatically represented in Figure 1.

### 2.1. LAR Subtype

First, the LAR subtype comprises heavily enriched hormonal regulation coupled with the absence of ER expression and displays overlapping patterns with luminal-type breast cancers [25]. The genetic alterations encountered in the LAR subtype comprise the biosynthetic processes of steroids, porphyrin metabolism, androgen/estrogen metabolism, and peroxisome proliferator-activated receptor (PPAR) signaling [26]. Phosphatidylinositol-4,5-bisphosphate 3-kinase catalytic subunit alpha gene (PIK3CA) gene mutations have been frequently recorded in patients with AR-positive TNBC [27]. This subtype is associated with apocrine histology, and AR has been identified as its surrogate biomarker, the mRNA levels of which are notably higher in LAR than in other TNBC subtypes [16]. The therapeutic strategy for the LAR subtype thus involves anti-androgen therapy, which diminishes the transcriptional signaling of androgen receptors [28]. Combinations of AR antagonists and phosphoinositide 3-kinase (PI3K) inhibitors have shown synergistic effects in AR-positive cell lines and xenograft models [29]. A recent approach involves the inhibition of Hsp90, a heat shock protein [30]. It has been identified as an ATP-dependent molecular chaperone that helps cancer cells maintain stability against cellular stress, the inhibition of which elevates cytotoxic effects and abrogates different tumorigenic pathways [31]. Compared to basal- and mesenchymal-like cell lines, these drugs have demonstrated higher efficacy in battling the LAR subtype [32].

### 2.2. M and MSL Subtype

The mesenchymal (M) and mesenchymal stem-like (MSL) subtypes are characterized by a cluster of genes linked to cell motility, cellular differentiation, and growth pathways [33]. The M subtype involves a stimulated transition of epithelial cells to mesenchymal cells (EMT) and activates mammary stem cell pathways. The MSL subtype, on the other hand, involves enhanced growth factor signaling when compared to the M type. It overexpresses genes associated with angiogenetic mechanisms and underexpresses claudin cluster genes (claudins-3, -4, -7, occludin, and E-cadherin) [34]. The histologic particulars of the mesenchymal subtype include sarcoma-like and squamous epithelial cell-like features and lymphocytic infiltration [35]. Anti-angiogenetic and TKI-inhibitory approaches involving drugs such as the vascular endothelial growth factor (VEGF)-neutralizing antibody bevacizumab and VEGF receptor tyrosine kinase inhibitors (sorafenib and sunitinib) are being used to mitigate mesenchymal-like TNBC [36]. It may also be sensitive to mTOR inhibitors because these cells express activated PI3K/AKT/mTOR signaling resulting from PIK3CA gene mutations [37].

### 2.3. Basal-like Subtype

A class of triple-negative cancer cells exhibits cytokeratins 5/17 and the EGFR/HER1, the genetic attributes of the basally situated epithelial layer of mammary glands, and defines the basal-line subtype [36]. The histology of basal-like tumors includes ductal carcinomas and invasive ductal carcinomas [38]. Significant activation of DNA damage and cell cycle response pathways is seen in the BL-1 type, which leads to enhanced cell proliferation. Thus, targeting DNA damage response pathways utilizing platinum-based therapies and polyadenosine diphosphate-ribose polymerase (PARP) inhibitors would serve as a rational therapeutic approach [39,40]. Embarking upon the BL-2 subtype TNBC, it involves enhanced signaling of growth factors, metabolic pathways, and overexpression of myoepithelial markers. The therapeutic strategy involves the administration of growth factor inhibitors targeting epidermal growth factor (EGF), mesenchymal epithelial transition factor receptor (MET), and insulin-like growth factor receptor (IGF1R) pathways alone or in combination with microtubule inhibitors [41].

### 2.4. Immunomodulatory Subtype

A special feature of the immune-enriched subtype is that it has overlapping features with the other subtypes. The immunomodulatory (IM) correlation represents the genetic expression caused by tumor-infiltrating lymphocytes, with signature tumor cells varying with subtype [42]. This correlation is encountered with the BL-1, BL-2, MSL, and LAR subtypes, excluding the M-classified tumors. The IM subtype involves genes involved in cytokine and immune signal transduction pathways exemplified by Th1/Th2, natural killer (NK) cells, B-cell receptor, dendritic cell (DC) pathway, T-cell receptor, interleukin (IL)-12, and IL-7 signaling pathways [25,43]. The histopathological findings showcase characteristics similar to medullary BC. Immune checkpoint inhibitors (ICIs), such as programmed cell death (PD-1), programmed cell death ligand-1 (PD-L1), and cytotoxic T-lymphocyte associated antigen-4 (CTLA-4), in combination with chemotherapeutic regimens have higher efficacy than ICI monotherapy in treating patients with IM subtype BC [44,45].

### 2.5. Basal-like and Immunosuppressed Subtype (BLIS)

The BLIS subtype involves abnormalities in DNA repair, regulation and replication processes. Moreover, BLIS showcases a dysfunctional cell cycle (mitotic prometaphase and cell division events). BLIS constitutes a pathogenesis that implicates few genes to be excessively expressed. These genes embody the protein regulator of cytokinesis 1 (PRC1), the mitotic checkpoint serine/threonine-protein kinase budding uninhibited by benzimidazoles 1 (BUB1) and protein coding genes centromere protein F (CENPF), which contribute to the highly proliferative nature of BLIS [8]. It causes decreased regulation of the immune responses exemplified by T-cell signaling, dendritic cell chemotaxis, B-cell signaling and reduced activation of innate immune responses.

### 2.6. Basal-like Immune Activated (BLIA) Subtype

BLIA belongs to one of the subtypes of Burstein’s classification of 2015. This subtype portrays the best prognostic parameters when compared to the other three (LAR, MES and BLIS) types of Burstein’s classification. Amplification of the cyclin dependent kinase 1 (CDK)1 gene has been associated with the pathogenesis of BLIA. In contrast to the BLIS subtype, BLIA shows upregulation of B cells, T cells and NK cells. Conversely, the genes involved in the STAT pathway are highly expressed [46].

## 3. The Biological Role of Androgen Receptor

The androgen receptor is a member of the steroid nuclear receptor family, accompanied by ER and PR. Being expressed in nearly 70–90% of invasive breast carcinomas, it is considered a nuclear hormone receptor with the greatest expression in BC [47]. The prevalence of AR expression in TNBC varies significantly in the cited literature, ranging between 6.6–75%. This wider range is applicable to the fact that there is heterogeneity among the reported studies with the number of patients enrolled and the cutoff values utilized to evaluate AR positivity [48]. Apart from its role in mammary cell proliferation and normal breast development, AR also plays a crucial role in cell signaling pathways [13].

**Figure 2:**
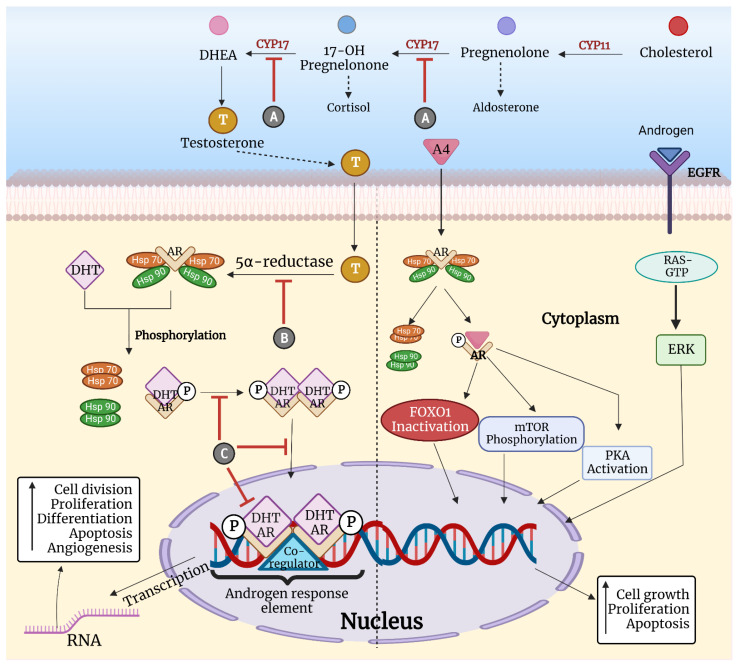
**Comprehensive signaling tactics ensuing the activation of androgen receptors along with targeting drug molecules.** A: CYP17 inhibitors (Abiraterone, Seviteronel, Orteronel). B: 5α-reductase inhibitors (dutasteride and finasteride). C: Androgen receptor antagonists (enzalutamide, bicalutamide, apalutamide, darolutamide, seviteronel, enobosarm, CR1447). Cytochrome P450 (CYP), Dehydroepiandrosterone (DHEA), Androgen receptor (AR), Heat shock protein (HSP), Ribonucleic acid (RNA), Forkhead box protein O1 (FOXO1), Mammalian target of rapamycin (mTOR), Protein kinase A (PKA), Extracellular signal-regulated kinase (ERK), Ras active guanosine triphosphate (RAS GTP). Testosterone (T), Dihydrotestosterone (DHT), Androgen receptor (AR), Androgen (A), Bicalutamide (Bic).

In the absence of a ligand, AR is bound to heat shock proteins (HSPs) in the cytoplasm, which stabilizes it by exposing the C-terminal ligand binding domain [49]. Upon its activation by circulating androgens, namely, testosterone (T) and dihydrotestosterone (DHT), homodimerization of the receptor takes place, which is followed by its translocation into the nucleus. In the nucleus, it binds to androgen response elements (AREs) with the subsequent activation and transcription of various downstream genes (KLK3, AZGP1, and PIP) [50,51]. Nevertheless, some nontranscriptional/nongenomic mechanisms, such as extracellular signal-regulated kinase (ERK)-dependent or ERK-independent signal transduction, may lead to AR activation without the need for DNA or RNA interactions. ERK-mediated AR signaling entails cytoplasmic AR that interacts with PI3K and Src proteins as well as Ras guanosine triphosphatase (GTPase) [52]. Non-ERK-mediated AR signaling requires the phosphorylation of mTOR, inactivation of forkhead box protein O1 (FOXO1) and activation of protein kinase A (PKA), which results in increased cell proliferation [53]. Comprehensive signaling tactics ensuring the activation of androgen receptors are diagrammatically represented in Figure 2.

Several preclinical studies have demonstrated that androgen signaling pathways play a crucial role in the development of malignant breast tissue, with some models implying their role in the progression of breast carcinoma [48]. AR also plays an important role in the development of metastasis by promoting migration and invasion via extracellular matrix degradation [54]. Studies analyzing the relationship between AR expression and clinicopathological features have yielded controversial results [55]. A recent study analyzing the correlations between AR expression and age revealed higher levels of AR tumors in older and postmenopausal patients than in younger and premenopausal patients [56]. Muller et al. observed that in LAR-TNBC, the lesions typically appear as spiculated margins (24.3% vs. 0–4.1% in other subtypes) or mammographic calcifications (8.1%) [57,58]. AR-positive tumors are highly differentiated tumors with low Ki-67 expression that display an irregular shape, which accounts for their low proliferation rate [59]. The literature disclosing the prognostic significance of AR in TNBC is not uniform, as great controversy exists regarding the effect of AR signaling in TNBC. The antiproliferative effects of AR signaling suggest a favorable prognosis associated with lower histologic grade, lower clinical stage and lower mitotic score for the LAR subtype [60]. Women with AR-positive TNBC reportedly had considerably better disease-free survival (DFS) and better overall survival (OS), according to Thike et al. These tumors also have a lower chance of recurrence, in contrast to AR-negative tumors, which are more likely to metastasize and reoccur [61]. On the other hand, some studies have suggested that AR expression is associated with higher mortality rates in patients with TNBC [14].

Androgen receptors can play a dual role in either suppressing tumors or stimulating the oncogenic elements of breast tumors. As a result, it can be established that AR agonists and antagonists can both play a substantial role in mitigating tumors depending on the presence of ERs and subtype of TNBC. If the tumors are both AR- and ER-positive, ARs become capable of competing with ERs to bind with estrogen-related elements. This eventually impairs ER transcription. However, in the case of AR-positive and ER-negative tumors, as seen with typical LAR TNBC, in the absence of ER competition, AR solely binds to androgen-responsive elements, which leads to the strengthening of tumorigenic characteristics and proliferation. This statement deduces the fact that in LAR-positive tumors, AR antagonists work efficiently and provide optimal results in comparison to AR agonists [62].

Additionally, selective androgen receptor modulators (SARMs) are the molecules that can elicit differing degrees of agonistic and antagonistic activity on AR, varying with the tissues. One such SARM, named Enobosarm, has been utilized in LAR-typical TNBC [63].

Several drug molecules have been developed to inhibit the binding of AR to androgens and the activation of AR because of their role in carcinogenesis. The encouraging results from targeting AR in prostate cancer have provided proof of concept for its application in breast cancers, including TNBC [64]. Androgen deprivation therapy in the form of 5α-reductase inhibitors (dutasteride, finasteride) and CYP17 inhibitors (abiraterone, seviteronel, orteronel) leads to a reduction in androgen biosynthesis, causing a decrease in the number of androgens to stimulate the signaling cascade [65,66]. Antiandrogens, such as AR antagonists, are a class of drugs that are nonsteroidal competitive inhibitors of AR that impede binding and nuclear signaling. enzalutamide, apalutamide, darolutamide, and abiraterone acetate are second-generation anti-androgens that provide the additional benefits of greater anti-proliferative effects, higher affinity, and repression of nuclear translocation [67,68]. Additionally, some studies pointed toward a greater frequency of *PIK3CA* mutations in AR+ TNBC, which led to the notion of combining PI3K inhibitors or dual PI3K/mTOR inhibitors along with anti-androgenic therapy [29]. Several preclinical models have demonstrated the synergistic activity of the mTOR inhibitors everolimus, trastuzumab, and enzalutamide to impede cell proliferation in AR+ ve TNBC [69]. Several mouse xenograft models and AR+ TNBC cell lines have shown that suppressing the PI3K/mTOR pathway leads to decreased AR expression [70]. Moreover, concurrent therapy with EGFR, Erk1/2 inhibitor, or PI3K inhibitor has been shown to hinder cell proliferation in TNBC cell lines [71]. The mechanistic workings of anti-androgenic drugs are graphically represented in Figure 2.

## 4. Substantiations Procured from the Clinical Trials

Several novel antiandrogenic agents are currently under investigation in AR-positive TNBC tumors. Clinical evidence suggests a role for anti-androgen therapies that offer an intriguing chemo-free alternative and therefore potentially shift current treatment strategies. Henceforth, we present evidence procured from several clinical trials evaluating anti-androgenic therapy in AR+ TNBC.

### 4.1. Seviteronel (SEVI)

SEVI (INO-464) has been identified to competitively antagonize androgen receptors and selectively inhibit the intratumoral androgen synthetic actions of cytochrome P45017a (CYP17) lyase [72]. This drug thus retards the formation of androgens in tumor cells along with simultaneous inhibition of AR binding [73].

It is a novel nonsteroidal agent that possesses the ability to sensitize AR+ TNBC models. An open-label, phase I/II clinical trial (NCT02580448) evaluated the clinical profile of SEVI in patients with TNBC or ER+/HER2 normal unresectable locally advanced BC. The goal was to determine the safety, efficacy, pharmacodynamic and pharmacokinetic parameters of SEVI. The phase I study included female participants, while phase II enrolled both men and women in their respective cohorts. Of the 175 patients anticipated, 13 patients were suitable to receive SEVI for phase 1. The primary objective of the phase 1 study was to establish the dose recommendations for phase 2 in female participants with BC. The single-dose Cmax was 17.4 ± 4.4 vs. 14.9 ± 4.3 µM, and the area under the curve (AUC) 0–8 h was 99.8 ± 17.9 vs. 78.9 ± 14.1 µM*h at 750 and 600 mg, respectively. These data interpret the dose of 750 mg to be more efficacious. The most common adverse events (AEs) associated with SEVI included fatigue, tremor, and vomiting along with a dose-limiting toxicity (DLT) of confusion, a Grade 3-related AE reported at 750 mg. The drug was found to be well tolerated with similar AE profiles in men and women [74]. SEVI, with a demonstration of encouraging results concerning the safety profile, has a dual mechanism of AR antagonism and androgen deprivation and might provide a novel treatment strategy to combat AR+ TNBC.

### 4.2. Enobosarm

Enobosarm, also known as ostarine, MK-2866 or GTx-024, is again a nonsteroidal SARM that serves to inhibit the growth of tumors. An open-label, phase II study (NCT02971761) was designed to assess the safety particulars of enobosarm plus pembrolizumab in addition to the determination of its response rate. Of 18 patients enrolled with a median age of 64 years, 16 patients were chosen for analysis of responses. The results showed a recurrence rate (RR) of 13%, clinical benefit response (CBR) of 25%, progression-free survival (PFS) of 2.6 months, and OS of 25.5 months. This combination, with a few grade 3 AEs comprising dry skin, diarrhea, and musculoskeletal ache, was well tolerated [75]. These results guide us toward the conclusion that combining AR modulators with ICIs could provide synergistic benefits and aid in the mitigation of TNBC. Nevertheless, future clinical trials assessing this combination are awaited.

Another phase II, open-label, multicenter and multinational investigational study enrolling patients (n = 32) was coordinated to determine the safety and efficacy endpoints of enobosarm in patients with AR+ TNBC (NCT02368691). The primary outcome to be evaluated was the CBR, while secondary outcomes included the CBR, best overall response, PFS, time-to-progression, duration of response (DoR), and overall response rate (ORR). This trial was terminated due to a lack of efficacy [76].

### 4.3. Bicalutamide

Bicalutamide is a nonsteroidal antiandrogen that competitively inhibits the binding of androgens with AR. It downregulates the expression of matrix metalloprotease-2 (MMP-2), matrix metalloprotease-9 (MMP-9) and cyclin D1. It can also induce cell cycle arrest at the G0/G1 phase [77]. An open-label, single-arm study (NCT02353988) was conducted to probe the role of bicalutamide in AR+ TNBC. Of the 424 patients enrolled, only 12% were reported to be AR+ and were included in the study. The results conveyed a median PFS of 12 weeks and a 6-month CBR of 19%. Bicalutamide was well tolerated with no presentation of grade 4/5 treatment-related AEs. In a selected group of patients with ER/PgR-negative and AR-positive BC, bicalutamide showed the efficacy of a minimally toxic androgen blockade [78].

A phase I/II single-arm, open-label, nonrandomized study (NCT03090165) evaluating the combination of bicalutamide and the CDK4/6 inhibitor ribociclib is under investigation in patients with advanced AR+ TNBC to assess the safety and tolerability profile of the combination [79].

Another ongoing study (NCT02605486) investigated bicalutamide in combination with the CDK4/6 inhibitor palbociclib in AR+ MBC patients to ensure the safety and tolerability of this combination [80]. Bicalutamide has provided us with a minimally toxic seandrogen blockade in AR+ tumors, and combining this molecule with CDK 4/6 inhibitors might lead us toward a novel combination that might expand the arsenal of treatment strategies to battle AR+ TNBC.

### 4.4. Enzalutamide

Enzalutamide is a second-generation nonsteroidal AR inhibitor that affects multiple steps in the AR signaling pathway, including the inhibition of translocation, cofactor assembly and ultimate transcriptional actions [81]. A phase II, single-arm, open-label, multicenter study (NCT01889238) evaluating the clinical activity and safety of enzalutamide in patients with advanced AR+ TNBC was conducted. Of the 118 enrolled patients, 78 patients were evaluable. The primary outcome included was CBR at 16 weeks, while the secondary outcomes included CBR at 24 weeks, PFS, and safety. Endpoints were analyzed among 2 groups: the intent-to-treat (ITT) population and the evaluable subgroup (ES) (tumor expressed ≥10% nuclear AR). The results obtained were that the CBR at 16 weeks was 25% in the ITT population and 33% in the ES population, the median PFS was 2.9 months in the ITT population and 3.3 months in the ES population, and the median OS was 12.7 months in the ITT population and 17.6 months in the ES population. Fatigue was the only treatment-related grade 3 adverse event, with an incidence of >2%. Enzalutamide demonstrated clinical activity and was well tolerated in patients with advanced AR-positive TNBC. This study supports the additional development of enzalutamide in advanced TNBC [19].

An open-label, randomized, phase Ib/2 trial (NCT02457910) of taselisib (GDC-0032), a PI3K inhibitor, in combination with enzalutamide in patients with AR+ TNBC was conducted to evaluate the side effects and best dose when given in combination and to evaluate the safety and efficacy of this combination. The primary endpoint was CBR at 16 weeks. The results showed a CBR of 35.7% and PFS of 3.4 months. Adverse events included hyperglycemia and skin rash. This trial was terminated due to an interim analysis of toxicity [29].

### 4.5. Abiraterone Acetate

Abiraterone significantly reduces androgen production by inhibiting cytochrome P450 17 alpha-hydroxylase (CYP-17), an enzyme that plays a substantial role in androgen biosynthetic mechanisms of tumor cells [82].

A phase II trial (NCT01842321) has been conducted evaluating the activity of abiraterone acetate in combination with prednisone in patients with molecular apocrine HER2-negative locally advanced or metastatic BC. Thirty-four patients with AR+ TNBC were included in the study. The primary endpoint, including the CBR at 6 months, was considered, and the secondary endpoints included the ORR, PFS, and safety. Treatment was proven to be well tolerated in some patients. The results comprised a CBR of 20%, an ORR of 6.7%, and a median PFS of 2.8 months. Fatigue, hypertension (HTN), hypokalemia, and nausea were some of the commonly found grade 1 or 2 drug-related AEs [83].

An ongoing trial (NCT04726332) of abiraterone acetate with fulvestrant, prednisone, and XL-102 is currently under investigation. It is a phase 1, open-label, dose-escalation and expansion study that aims to evaluate the safety, tolerability, pharmacokinetics, antitumor activity, and effects of this agent on biomarkers, alone and in multiple combination regimens, in subjects with advanced solid tumors [84].

### 4.6. Orteronel

Orteronel is a novel, orally administered, selective nonsteroidal inhibitor of 17,20-lyase, an essential enzyme in the biosynthetic pathway of androgens. The agent is under clinical evaluation as a potential therapeutic strategy against hormone-sensitive cancers. A phase II study has been established in patients with AR-positive triple-negative tumor cells who have previously been treated with standard therapeutic regimens to evaluate its androgen blocking activity. Twenty-six patients were enrolled with a median age of 57 years. The results showed that the ORR was 4%, the disease control rate (DCR) was 15%, the median PFS was 2 months, and the median OS was 10.2 months. Nausea and fatigue were the most frequently reported AEs. Grade 3/4 AEs comprised HTN, increased amylase, and lipase. Additionally, four patients reported serious adverse events (SAEs) of pneumonitis, chest pain, peripheral edema and prolonged QT under the Grade 2 category with hypokalemia of the Grade 4 category. The treatment regimen was assessed to be well tolerated, notwithstanding the limited clinical activity in the previously treated AR+ TNBC patients. However, the trial closed early due to slow accrual. Of all patients who discontinued treatment, 85% were due to disease progression, and 15% were due to AEs [85].

### 4.7. CR1447

Fluoxymesterone, also known as CR1447 or 4-hydroxytestosterone (4-OHT), is an androgen receptor modulator that exerts antiproliferative effects in ER + ve and ER-ve/AR+ ve breast cancer cells. It is a small molecule that has two distinct properties. One is the steroidal aromatase inhibitor (AI), and the other property is its capability to bind to the AR with higher affinity. This SARM has the advantage of not causing significant hirsutism, in contrast to the testosterone used for treating AR-positive BC [86].

### 4.8. Finasteride and Dutasteride

Finasteride and dutasteride are small molecule inhibitors that block the 5alpha reductase enzyme, resulting in decreased conversion of testosterone to dihydrotestosterone [87]. However, finasteride is contraindicated in women of child-bearing age. At present, no clinical studies are under investigation for the use of these agents in AR+ ve TNBC.

To conclude, the substantiation of targeting AR has opened doors to broadening the treatment strategies for AR+ TNBC with encouraging results from clinical trials. As the only side effects encountered are nausea and fatigue, anti-androgenic monotherapy has been beneficial if the prevention of toxicity is prioritized (exception being ortorenol).

The combinations of AR antagonists with the immunotherapeutic agents pembrolizumab and prednisone have shown enhanced efficacy but with a rise in grade 2 and 3 events. Bicalutamide and seviteronel have exhibited remarkable safety profiles along with improvement in the survival of patients. Further studies are being conducted to investigate the combination of AR-targeting therapies with targeted chemotherapeutic therapies such as CDK inhibitors and PI3K inhibitors along with ICIs. Several completed and ongoing clinical trials assessing anti-androgens in AR+ TNBC are summarized in Table 1 and Table 2.

## 5. Conclusions

AR-positive TNBC presents a dismal prognosis with a higher mortality rate and a challenging clinical course. The conventional chemotherapeutic regimens had a paucity of requisite clinical responses, and this demanded the discovery of a novel standpoint in combating the formidable battle against LAR typical TNBC. With this emerged the idea of targeting the ARs, which showcased substantial expression in luminal triple-negative cells. Androgen receptors have been established to receive stimulations from androgens and support the mediation of hormonal regulation in carcinogenetic events. Despite the demonstration of encouraging efficacy endpoints by a plethora of clinical trials assessing anti-androgenic agents, the data still exhibit controversies. Although approaches utilizing bicalutamide and seviteronel have shown promising results, few studies evaluating this class of drugs have evidenced the shortcomings of unsatisfactory efficacy, notable disease progression, and adverse event profiles. Anti-androgenic drugs in combination with CDK inhibitors, PI3K inhibitors and ICIs are being assessed and anticipate a propitious response. Improvements are being witnessed in formulating these drugs, and newer anti-androgens with exceptional properties can be expected in the near future. This lends us to the conclusion that the anti-androgenic approach is capable of paving an optimistic path against LAR-TNBC.

## Figures and Tables

**Table 1:** List of completed trials presenting the clinical statistics of anti-androgenic drugs.

Drug	No. of Patient	Phase	Patient Cohort	Regimen	Comparator Arm	Result	Comment	Common Toxicity	NCT No.
Seviteronel	175	I/II	TNBC or ER +/HER2 normal unresectable locally advanced breast cancer.	Seviteronel	Seviteronel at 650 mg and 700 mg.	Cmax: 750 mg: 17.4 ± 4.4 µM vs. 600 mg: 14.9 ± 4.3 µM. AUC: 750 mg: 99.8 ± 17.9 µM*h vs. 600 mg: 78.9 ± 14.1 µM*h.	Sevi was well tolerated with an adverse event profile similar to that in men.	Fatigue, tremor, and vomiting.	NCT02580448
Enobosarm	18	II	Androgen receptor+ mTNBC.	Pembrolizumab and Enobosarm	-	ORR: 13%, Median PFS: 2.6 months. CBR: 25%. Median OS: 25.5 months.	The combination of enobosarm and pembrolizumab was well tolerated in heavily pretreated AR+ TNBC.	Grade 3 adverse events: dry skin, diarrhea, and musculoskeletal ache.	NCT02971761
Bicalutamide	60	II	AR+ Metastatic TNBC	Bicalutamide and other according to physician’s choice.		Median PFS: 12 weeks. CBR: 19%	Bicalutamide shows efficacy in a select group of patients with ER/PR-negative, AR-positive breast cancer.	Bicalutamide was well-tolerated with no grade 4/5 treatment-related adverse events.	NCT02353988
Enzalutamide	118	II	Advanced, AR+, TNBC.	Enzalutamide	ITT population and >10% nuclear AR (evaluable subgroup).	Median PFS: ITT: 2.9 months and ES: 3.3 months. CBR: ITT: 25% and ES: 33% at 16 weeks. Median OS: ITT: 12.7 months and ES: 17.6 months.	Enzalutamide was well tolerated in patients with advanced AR-positive TNBC.	Fatigue (>2%).	NCT01889238
Abiraterone acetate	34	II	Molecular apocrine HER2-negative locally advanced or metastatic breast cancer	Abiraterone acetate plus prednisone		ORR: 6.70%. Median PFS: 2.8 months. CBR: 20% at 6 months.	Treatment was found to be beneficial for some patients.	Fatigue, HTN, Hypokalemia, and nausea were commonly found AE and were grade 1 or 2.	NCT01842321
Orteronel	26	II	AR expressing metastatic TNBC previously treated with standard therapy.	Orteronel		ORR: 4%. Median PFS: 2.0 months. CBR: 15%. Median OS: 10.2 months	Treatment was well tolerated but showed limited clinical activity in previously treated patients with TNBC.	Common: Nausea and fatigue. Grade 3/4: HTN, increased amylase and lipase. SAEs seen in 4 patients: G2 pneumonitis, G2 chest pain, G2 peripheral edema, G4 prolonged QT, and G4 hypokalemia.	NCT01990209

TNBC-Triple Negative Breast Cancer, ER-Estrogen Receptor, HER2-human epidermal growth factor receptor 2, RR-Response Rate, PFS-Progression free Survival, CBR-Clinical benefit rate, OS-Overall Survival, AR-Androgen Receptor, RR-Response Rate, AUC-Area under curve, AE-Adverse Event, PgR-Progesterone Receptor, HTN-Hypertension, G4- Grade 4, G2- Grade 2, ITT-Intent-to-treat.

**Table 2:** List of ongoing trials aiming to evaluate the clinical profile of anti-androgenic drugs.

Drug	Phase	No. of Patients	Patient Cohort	Treatment Arm	Primary Outcomes	Secondary Outcomes	Status	NCT No.
AR inhibitor	Ib/II	140	LAR subtype without HER2 gene activated mutation.	AR inhibitor combined with everolimus (B1) or CDK4/6 inhibitor (B2), or EZH2 inhibitor (B4)	ORR	DOR, PFS, OS	Recruiting	NCT03805399
Enzalutamide	II	50	Early- Stage AR (+) TNBC	Enzalutamide	Treatment discontinuation rate	-	Active, not recruiting	NCT02750358
Bicalutamide	I/II	37	Advanced AR+ TNBC	Ribociclib, Bicalutamide	MTD, CBR	Phase1: ORR, DoR, safety and tolerability, PK parameters. Phase 2: PFS, ORR, and OS.	Recruiting	NCT03090165
Enzalutamide	II	37	AR+ TNBC.	Enzalutamide, Paclitaxel	Incidence of pCR, residual cancer burden-index, and minimal residual disease.	PFS	Recruiting	NCT02689427
Enzalutamide	I/II	246	Advanced solid tumors	NUV868 as monotherapy and in combination with Olaparib or Enzalutamide.	DLTs, PK, ORR, and PSA-RR		Recruiting	NCT05252390
Abiraterone	I	298	Locally advanced or metastatic solid tumors	Fulvestrant, Abiraterone, Prednisone	MTD, ORR	Incidence and severity of AEs, tolerability, drug-drug interactions, and PK parameters.	Recruiting	NCT04726332
Enzalutamide	II	221	Primary Breast Cancer (ARB) AR+ and ER+ TNBC.	Enzalutamide, alone or in combination with exemestane.	Geometric mean change and anti-proliferative response.	Geometric mean change, anti-proliferative response, safety, tolerability, and apoptotic response.	Unknown	NCT02676986
CR1447	II	29	HER2 negative and AR+ triple negative metastatic or locally advanced breast cancer.	CR1447	Disease control at 24 weeks	Adverse events, Pk analysis, disease control at 12 weeks, change in tumor size at 12 weeks, Ki67 expression, estradiol levels during treatment, and mRNA expression.	Active, not recruiting	NCT02067741
Darolutamide	II	94	Triple negative AR+ locally recurrent (Unresectable) or metastatic breast cancer.	Darolutamide + Capecitabine	CBR	CBR, ORR, DoR, OS, PFS, and evaluation of toxicity.	Active, not recruiting	NCT03383679
Bicalutamide	I/II	46	AR+ TNBC.	Palbociclib + Bicalutamide	Recommended phase II dose and PFS.	ORR, CBR, PFS, safety, and tolerability.	Active, not recruiting	NCT02605486
Bicalutamide	II	46	AR+ TNBC	Nivolumab, Ipilimumab, Bicalutamide	CBR at 24 weeks	PFS, OSR, ORR	Recruiting	NCT03650894

TNBC—triple-negative breast cancer, AR—androgen receptor, AR—androgen receptor, LAR—luminal androgen receptor, ORR—objective response rate, DLTs—dose-limiting toxicity, AEs—adverse events, ER—estrogen receptor, HER2—human epidermal growth factor receptor 2, DOR—duration of response, OS—overall survival, PK—pharmacokinetics, pCR—pathologic complete response, PSA-RR—prostate-specific antigen response rate, MTD—maximum tolerated dose, CBR—clinical benefit rate, CBR—clinical benefit rate.

## Data Availability

Not applicable.
